# Supplementary material for: PTEN Loss Enhances Error-Prone DSB Processing and Tumor Cell Radiosensitivity by Suppressing RAD51 Expression and Homologous Recombination
Source: Int J Mol Sci. 2022 Oct 25;23(21):12876. doi: 10.3390/ijms232112876 (PMC9658850; doi:10.3390/ijms232112876)
Supplement: Supplementary file 1 [file ijms-23-12876-s001.zip › MS-249-Pei-PTEN-Suppl-Information-2022-10-19.pdf]

Supplementary Information

PTEN loss enhances error-prone DSB processing and tumor cell radiosensitivity by suppressing RAD51 expression and homologous recombination

Xile Pei, Emil Mladenov, Aashish Soni, Fanghua Li, Martin Stuschke and George Iliakis

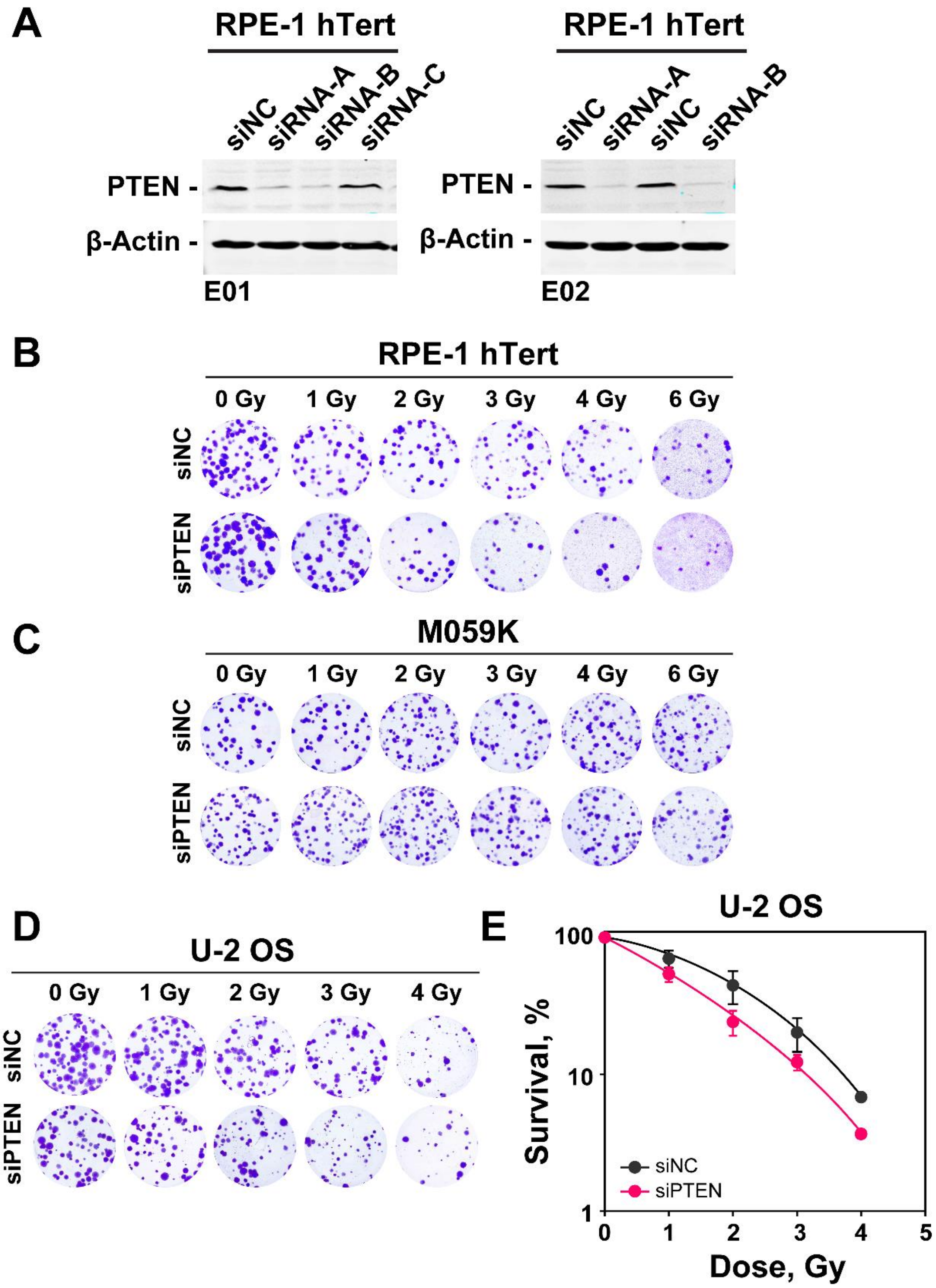

Figure S1. A) Western blot analysis of PTEN protein level in cells transfected with indicated PTEN specific siRNAs. Negative control siRNA (siNC) was used as control. **B and C)** Representative images of colonies obtained after crystal violet staining of dishes from clonogenic survival experiments quantified in Figure 1D. **D)** Representative images of colonies obtained after crystal violet staining of dishes utilized in clonogenic survival of U-2 OS cells. **E)** Clonogenic survival experiments of U-2 OS cells transfected or not with PTEN siRNA. Data represent the mean ± SD from three independent experiments.

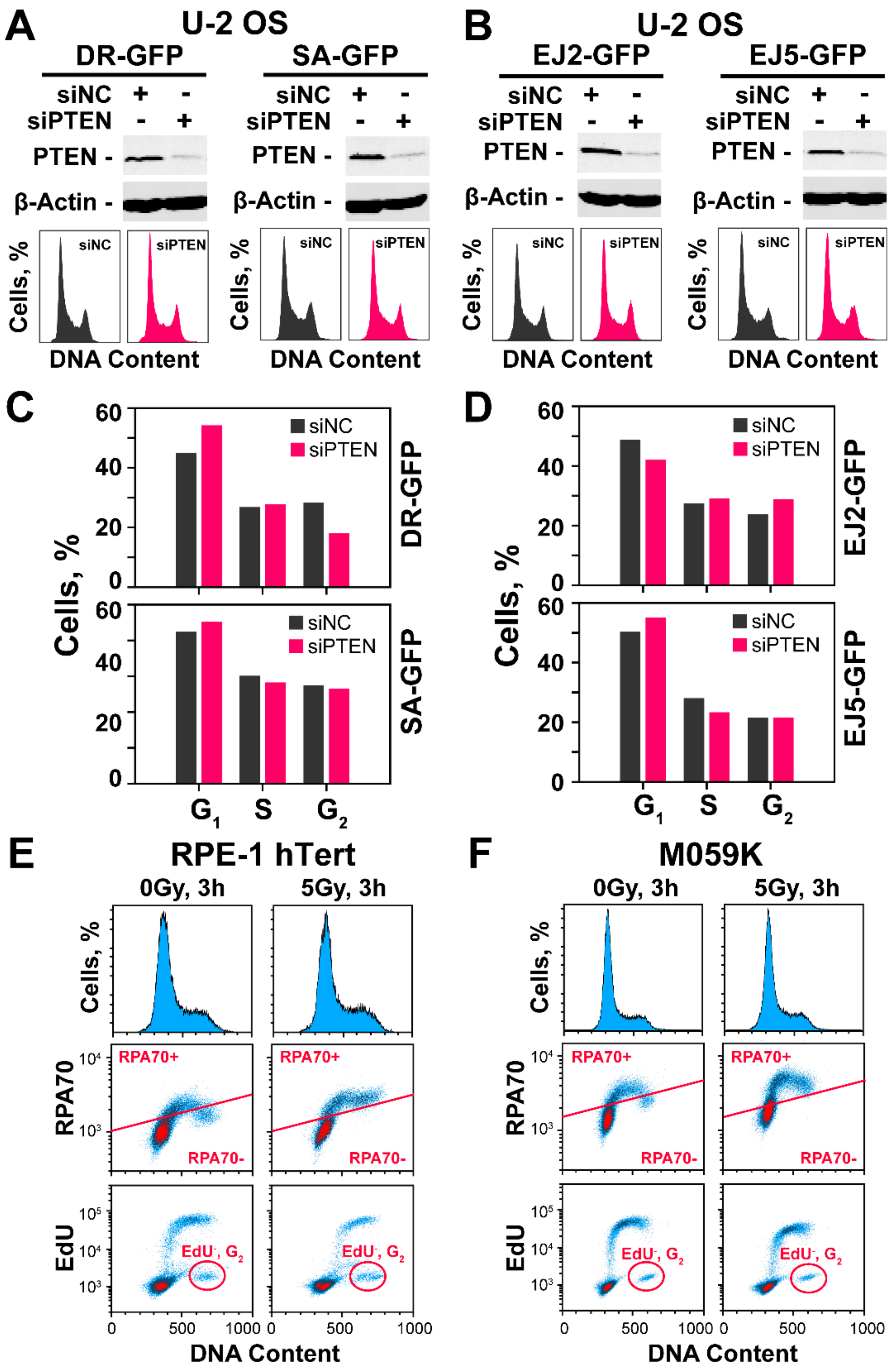

Figure S2. A and B) Western blot analysis of PTEN expression in different U-2 OS reporter cell lines transfected with siNC or siPTEN and measured 48 h after transfection. The cell cycle distribution of transfected cells was analyzed by flow cytometry. **C and D)** Representative FACS histograms of RPE-1 hTert and M059K cells, showing the EdU^{-} G_2-gating scheme used to measure resection by three parametric flow cytometry detecting DAPI, EdU and RPA70 signals, 1 h post irradiation.

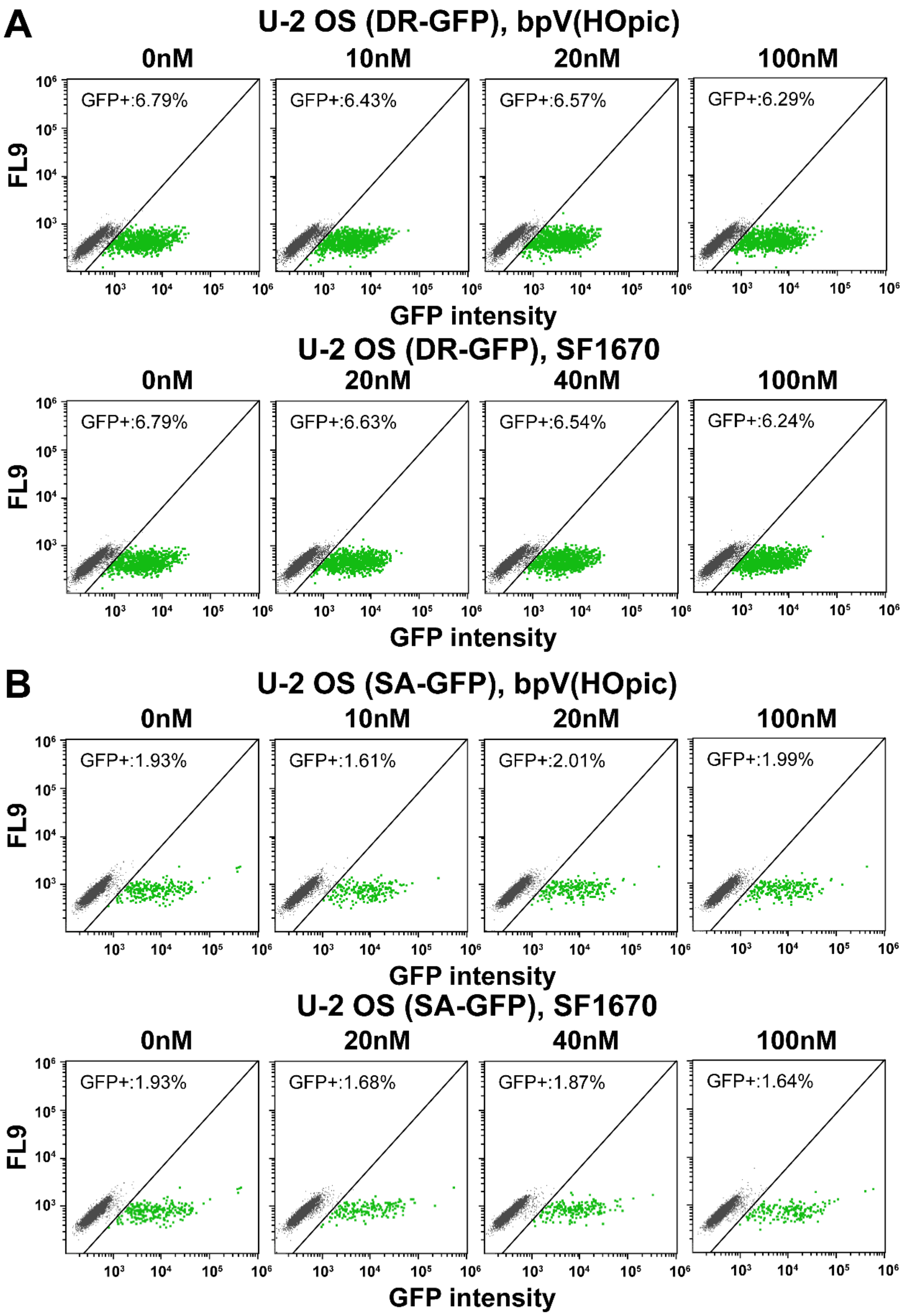

Figure S3. **A).** Representative dot plots of DR-GFP U-2 OS cells, treated with the PTEN inhibitors (bpV(HOpic) or SF1670) and utilized to generate the data presented in Figure 6B. **B).** Same as panel A), but for U-2 OS cells reflecting the repair of DSBs by SSA (SA-GFP).

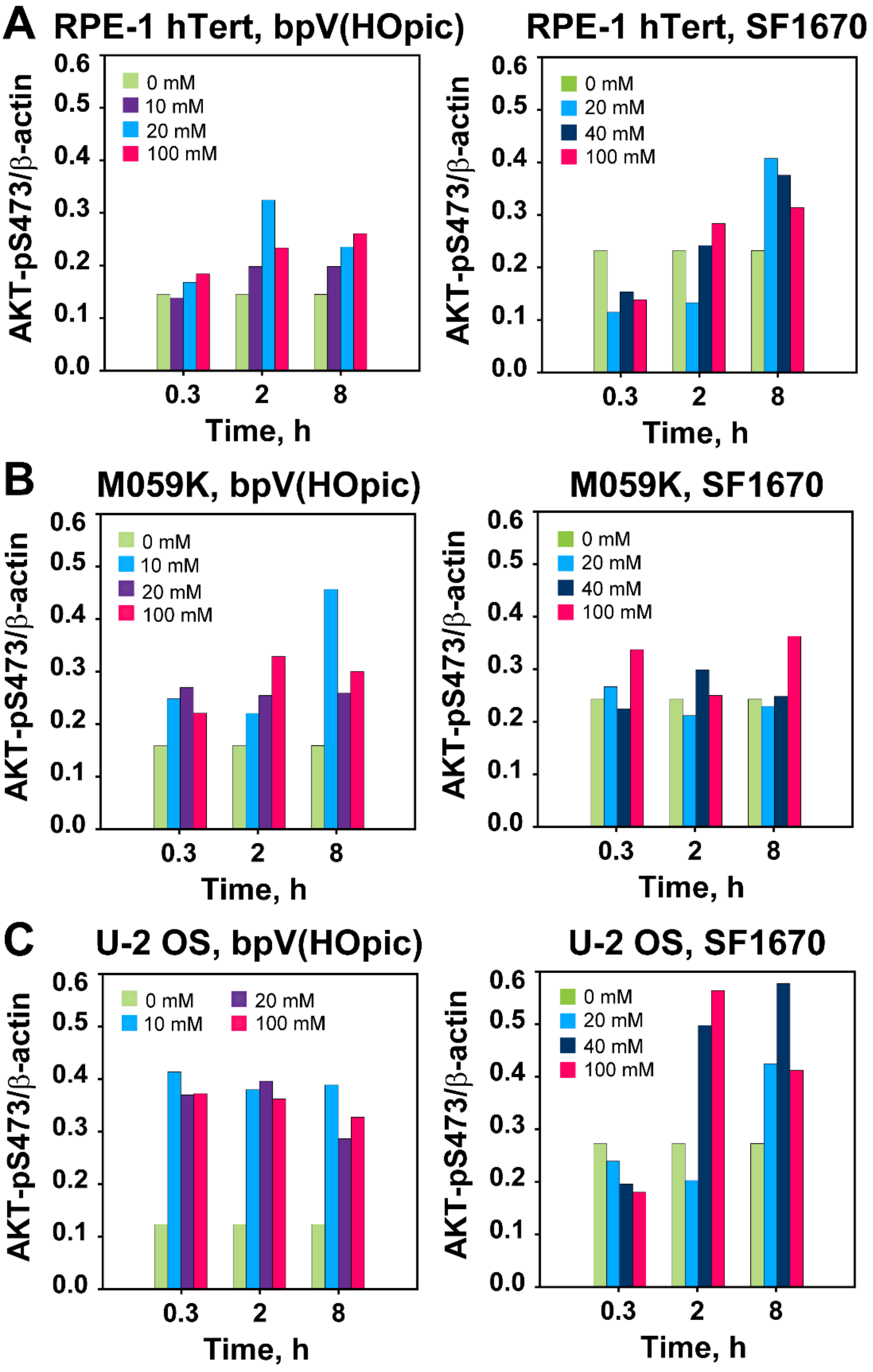

Figure S4. Densitometry analysis of western blots, presented in Figure 5C, 5D and 5E. For densitometry analysis a free version of the dedicated densitometry software QuantityOne, Version 4.6.2, Build 070 (BioRad) was utilized according to the operational manual. The intensity per mm^2 of every lane was evaluated after background subtraction. The corresponding intensity values for the AKT-pS473 were normalized against the values determined for the β-actin, loading control.

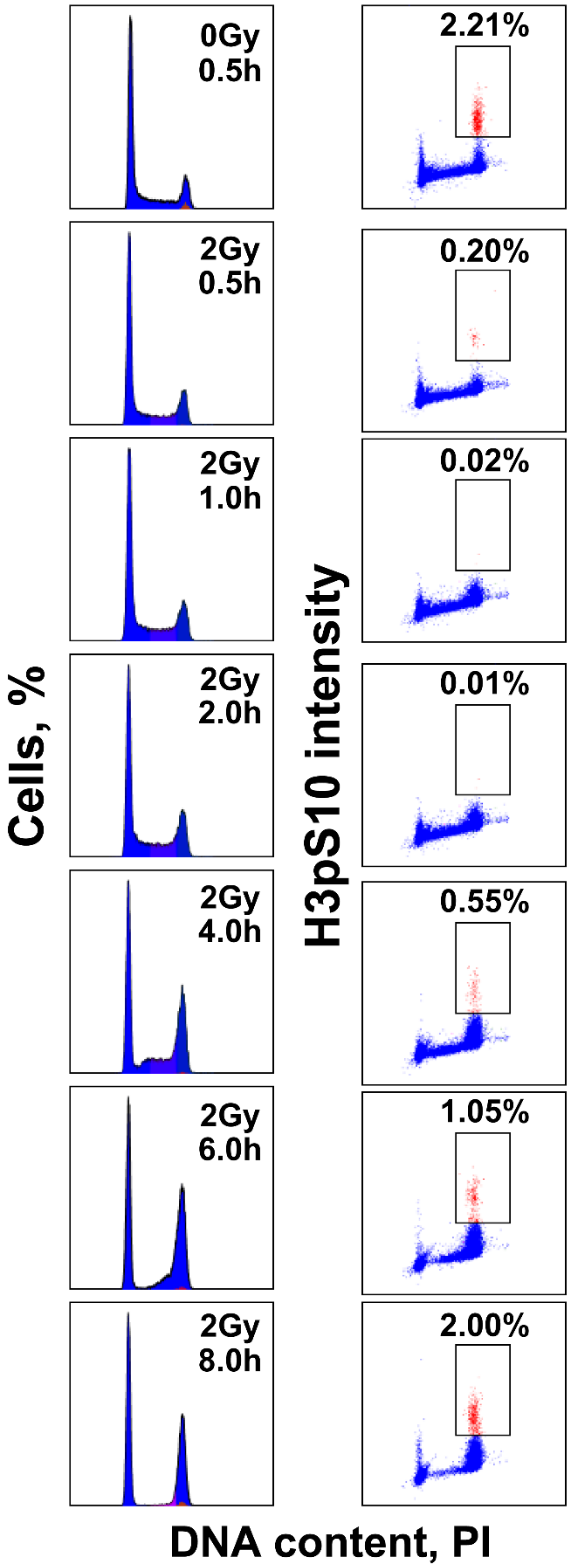

Figure S5. Representative histogram plots, showing the distribution of RPE-1 hTert cells throughout the cell cycle at different times after exposure to 2 Gy of x-rays (left row of panels). Representative dot plots of H3pS10 signal, indicating the number of cells in mitosis (see text for details).

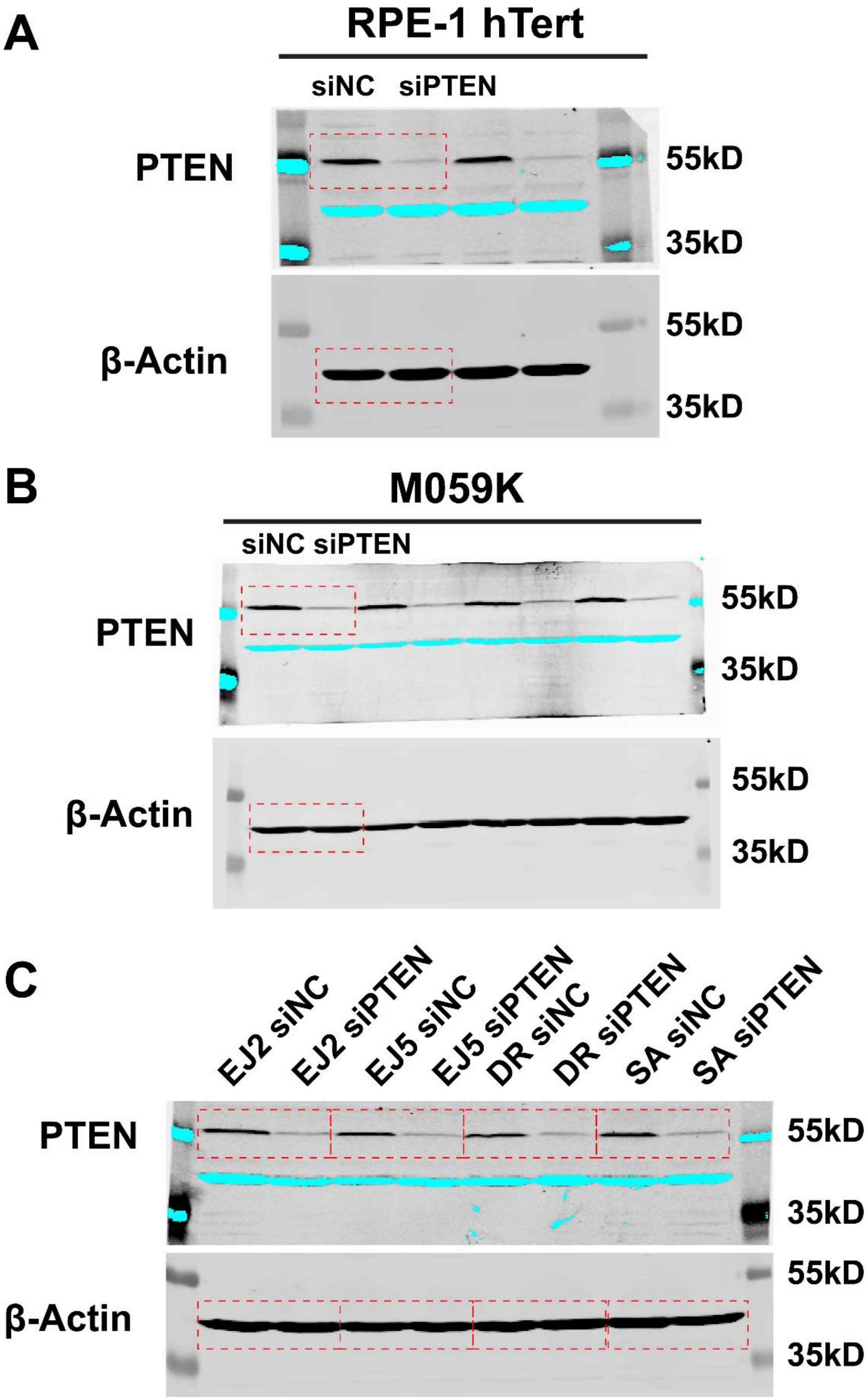

Figure S6. **A and B)** RAW, uncropped Western blot membranes corresponding to the analysis shown in Figure 1A. **C)** RAW, uncropped Western blot membranes corresponding to the analysis shown in Figure S2A and S2B.

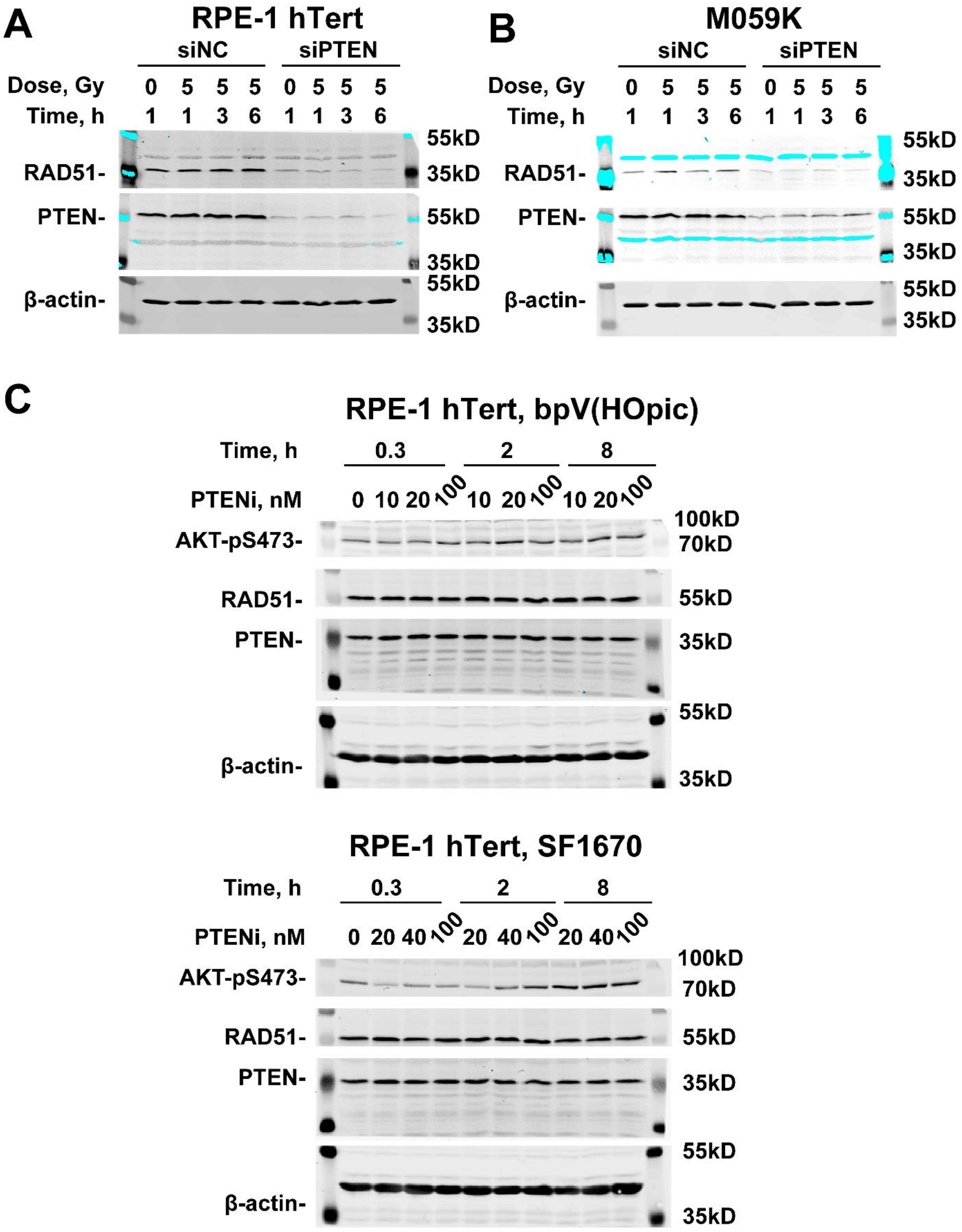

Figure S7. A and B) RAW, uncropped Western blot membranes corresponding to the analysis shown in Figure 5A and 5B. **C)** RAW, uncropped Western blot membranes corresponding to the analysis shown in Figure 5C.

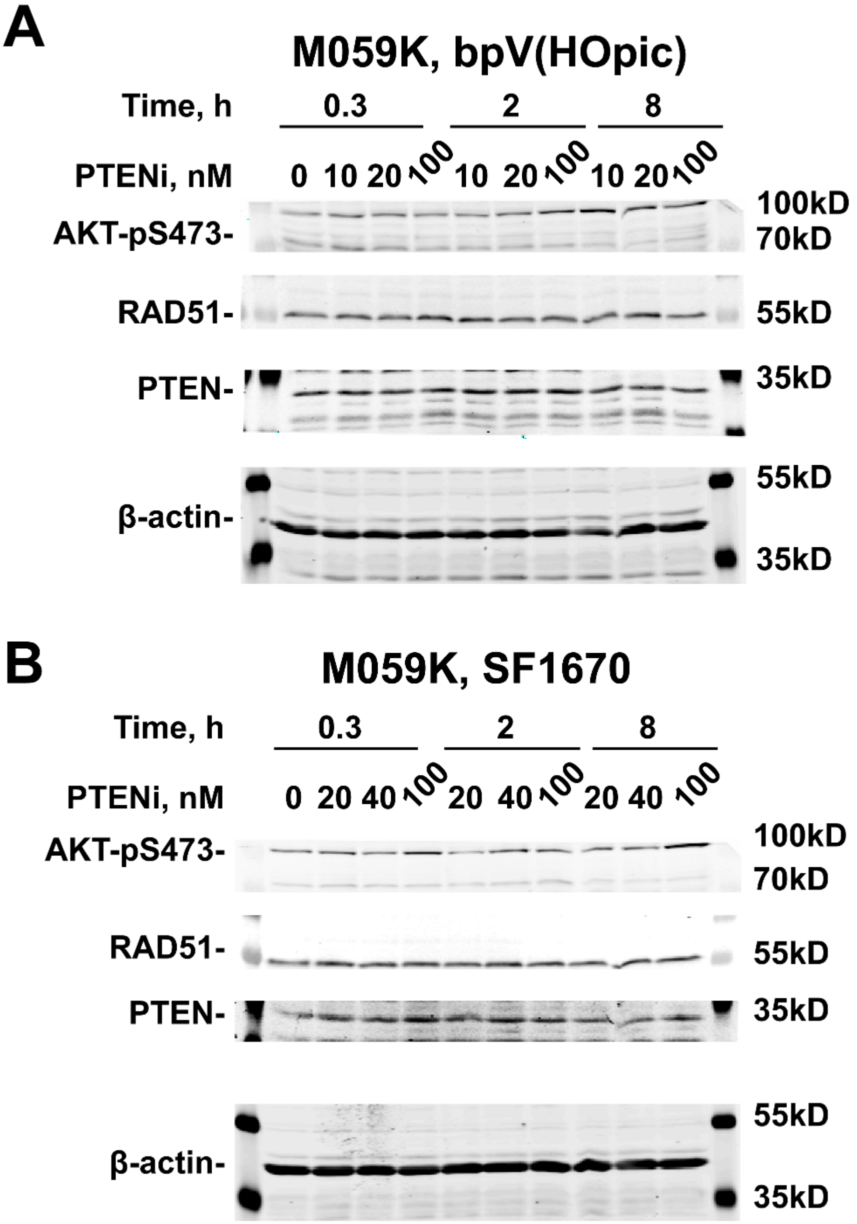

Figure S8. A and B) RAW, uncropped Western blot membranes corresponding to the analysis shown in Figure 5D.

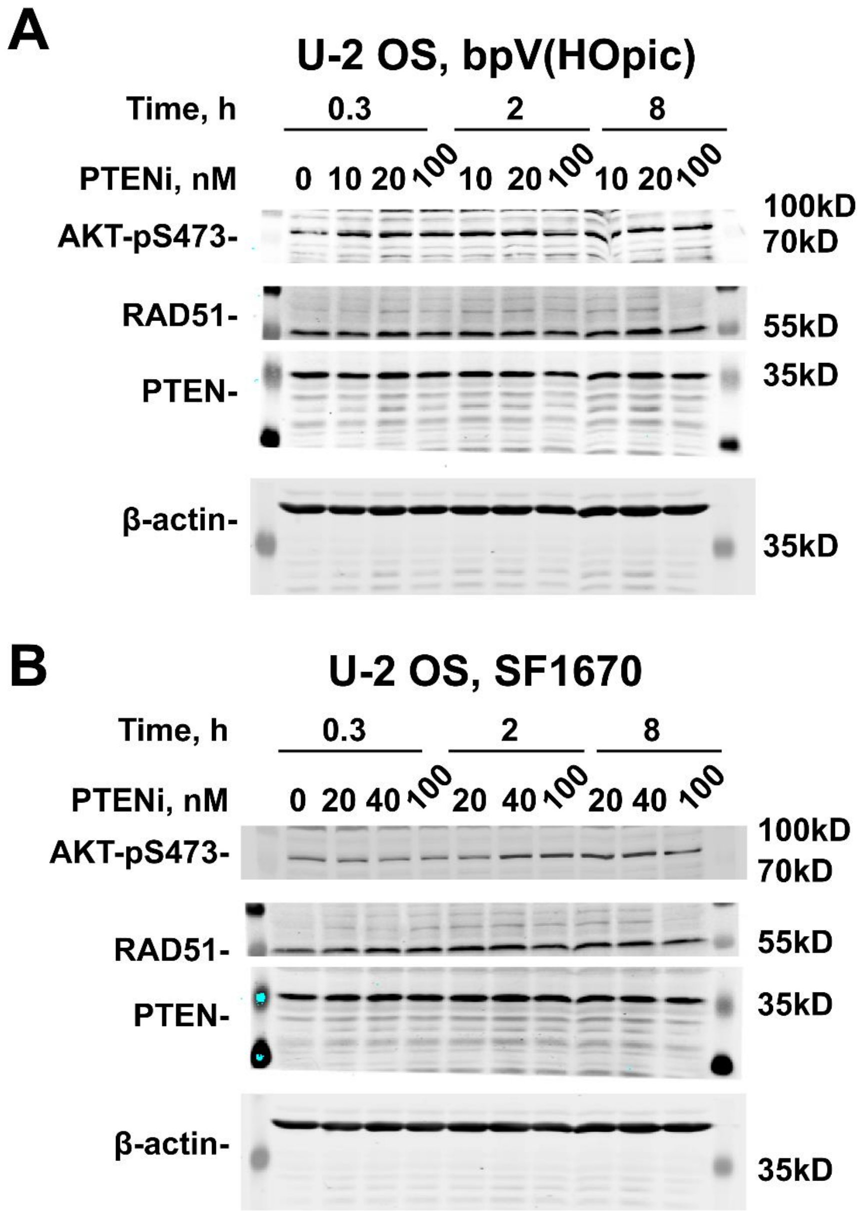

Figure S9. A and B) RAW, uncropped Western blot membranes corresponding to the analysis shown in Figure 6A.
